# Network Assessor: an automated method for quantitative assessment of a network's potential for gene function prediction

**DOI:** 10.3389/fgene.2014.00123

**Published:** 2014-05-16

**Authors:** Jason Montojo, Khalid Zuberi, Quentin Shao, Gary D. Bader, Quaid Morris

**Affiliations:** Donnelly Centre for Cellular and Biomolecular Research, University of TorontoToronto, ON, Canada

**Keywords:** network inference, function prediction, cross validation, network biology, machine learning

## Abstract

Significant effort has been invested in network-based gene function prediction algorithms based on the guilt by association (GBA) principle. Existing approaches for assessing prediction performance typically compute evaluation metrics, either averaged across all functions being considered, or strictly from properties of the network. Since the success of GBA algorithms depends on the specific function being predicted, evaluation metrics should instead be computed for each function. We describe a novel method for computing the usefulness of a network by measuring its impact on gene function cross validation prediction performance across all gene functions. We have implemented this in software called Network Assessor, and describe its use in the GeneMANIA (GM) quality control system. Network Assessor is part of the GM command line tools.

## Introduction

Networks of gene-gene functional interactions (or more generally, associations) have proven useful to predict gene function (Zhang et al., 2004; Mostafavi et al., 2008; Peña-Castillo et al., 2008). In this model, the nodes of the network are genes and the edges represent specific types of associations between them. For example, a gene can be connected to other genes that inhibit or promote it, that encode similar protein domains, that share similar expression profiles, that are located close together on the same chromosome, or whose products physically interact with its products.

Various methods exist for predicting function from gene-gene networks. The most common approach uses some variation of the guilt by association (GBA) principle (Schwikowski et al., 2000; Hishigaki et al., 2001; Wu et al., 2002; Vazquez et al., 2003; Deng et al., 2004; Ye et al., 2005; Sharan et al., 2007; Franceschini et al., 2013; Zuberi et al., 2013). This assumes the function of a gene can be inferred from its neighbors in the network by following edges. Guilt-free approaches also exist, such as using the node degree of a gene without considering any properties of its neighbors (Gillis and Pavlidis, 2011). These algorithms use binary classification to perform predictions for one function at a time. Multi-label classifiers also exist that can make predictions for multiple functions simultaneously (Wang et al., 2013; Yu et al., 2013).

Network-based gene function algorithms have demonstrated strong performance for multifunctional genes (Gillis and Pavlidis, 2011). Algorithms that use binary classification are typically evaluated by using cross validation against a gold standard, such as gene annotations from Gene Ontology (GO) (The Gene Ontology Consortium, 2000) or FunCat (Ruepp et al., 2004). This process involves passing the association networks and a subset of the genes in a specific GO term as input to a binary classifier, which implements a particular prediction algorithm. The classifier then attempts to recover the withheld genes by ranking them based on the likelihood that they are members of the GO term. From this ranking, the area under the receiver operating characteristic curve (AUROC) and the area under the precision-recall curve (AUPR) metrics are computed (Fawcett, 2006). The AUROC and AUPR values are typically aggregated across GO terms to produce a mean AUROC and mean AUPR. The input association networks may need to be integrated into a single graph prior to binary classification, depending on the prediction algorithm used. A similar process can be used with multi-label classifiers when evaluating label-based performance (Tsoumakas et al., 2010).

When the input association networks and gold standard are held constant, we can use this process to compare the performance of different prediction algorithms. However, if we instead fix the algorithm and gold standard, we can assess the usefulness of the input association networks for particular tasks, such as assigning gene membership to GO terms.

Quantifying the usefulness of specific gene-gene networks for function prediction is difficult in general. The topology of a network may impact prediction performance differently depending on the function in question. Sometimes a small fraction of edges may account for most of the cross validation performance for a large number of GO terms (Gillis and Pavlidis, 2012). Larger networks are more likely to include more of these informative edges, but it's also possible for a large network to have only a few of them. Similarly, a small network can be constructed to contain a large proportion of such critical or exceptional edges.

The software we present, Network Assessor, was designed for gene function-specific quantification of the usefulness of association networks for prediction tasks. In particular, the software quantifies the predictive potential of one or more networks by reporting the differences in cross validation performance for each GO term, with and without the network(s) in question. Although the software provides built-in support for using the latest GO annotations, any annotation set can be used as the gold standard. Network Assessor has already been used to demonstrate that genetic interaction networks obtained under different experimental conditions provide complementary information that improves gene function prediction performance (Michaut and Bader, 2012).

Network Assessor currently uses the GeneMANIA (GM) algorithm (Mostafavi et al., 2008), which is a fast, real-time network integrator and binary classifier that uses GBA to infer gene function. Recent studies have indicated that cross validation performance of GBA-based algorithms depends on the GO term being tested (Gillis and Pavlidis, 2012). Although Network Assessor uses a GBA-based predictor, it can be readily extended to use non-GBA algorithms and even multi-label classifiers. Network Assessor permits term-by-term analysis by providing AUROC and AUPR metrics for each GO term rather than averaging over all GO terms.

Network Assessor was originally used to analyze changes in prediction performance between different releases of the GM web server (Warde-Farley et al., 2010; Zuberi et al., 2013). The results of this analysis help identify issues with GM network data, make parameter decisions and are used to evaluate new networks for inclusion in the system.

### Experimental objectives

We designed Network Assessor to quantify the usefulness of an association network for predicting gene function. However, directly measuring the predictive potential of an arbitrary network in isolation by simply assessing the degree to which the association network connects nodes with similar labels is not necessarily informative because of the synergistic nature of network data. For example, suppose you have two non-overlapping networks, A and B, and another network C that overlaps with both A and B. Predictions that use only A, B, or C in isolation would be very different from those made using the integration of all three because the set of reachable neighbors in the latter is much larger. This difference is significant for predictors based on label propagation (Kato et al., 2009) that utilize indirect connections between genes. Our approach allows us to measure the impact of adding (or withholding) network C.

Since prediction performance may vary by GO term, Network Assessor computes the relative predictive potential of an association network by measuring the impact of adding it to (or removing it from) a network with known predictive potential for each GO term. This allows researchers to examine differences in performance by GO term size and position in the GO hierarchy.

### Limitations of current techniques

Alternative techniques for quantifying the usefulness of networks in gene function prediction exist. For instance, identifying which gene functions follow the GBA principle in a given network can be accomplished by applying statistics originally developed for testing spatial clustering in proximity networks (Kleessen et al., 2013). The degree of global spatial autocorrelation (such as Moran's I statistic) indicates whether gene expression correlates well with gene function. This is useful for investigating which gene functions follow the GBA principle. In contrast to these methods, Network Assessor measures the effect on prediction performance of an arbitrary network for all known gene functions one at a time.

Furthermore, Network Assessor provides a network integrator to allow the evaluation of sets of networks from different sources. This is critical for organisms with poor annotation coverage. Intra-species transfer of annotations and the integration of functional interaction networks derived through orthology would likely improve prediction performance (Klie et al., 2012).

In particular, Network Assessor also permits the analysis of different combinations of networks. It uses GM's various network integration algorithms to combine multiple weighted undirected networks into a single weighted undirected network. For example, the default behavior uses the Simultaneous Weights and Unregularized algorithms (Mostafavi and Morris, 2010) to assign weights to each network indicating its information content for the GO term prediction task at hand. This weight is multiplied with each of that network's edges during network integration. Weights can also be assigned equally by network.

Network Assessor makes analysis of association networks more accessible to both computational and non-computational scientists who otherwise must script their own analysis or do not have access to automated tools, respectively.

## Network assessor

**Figure 1:**
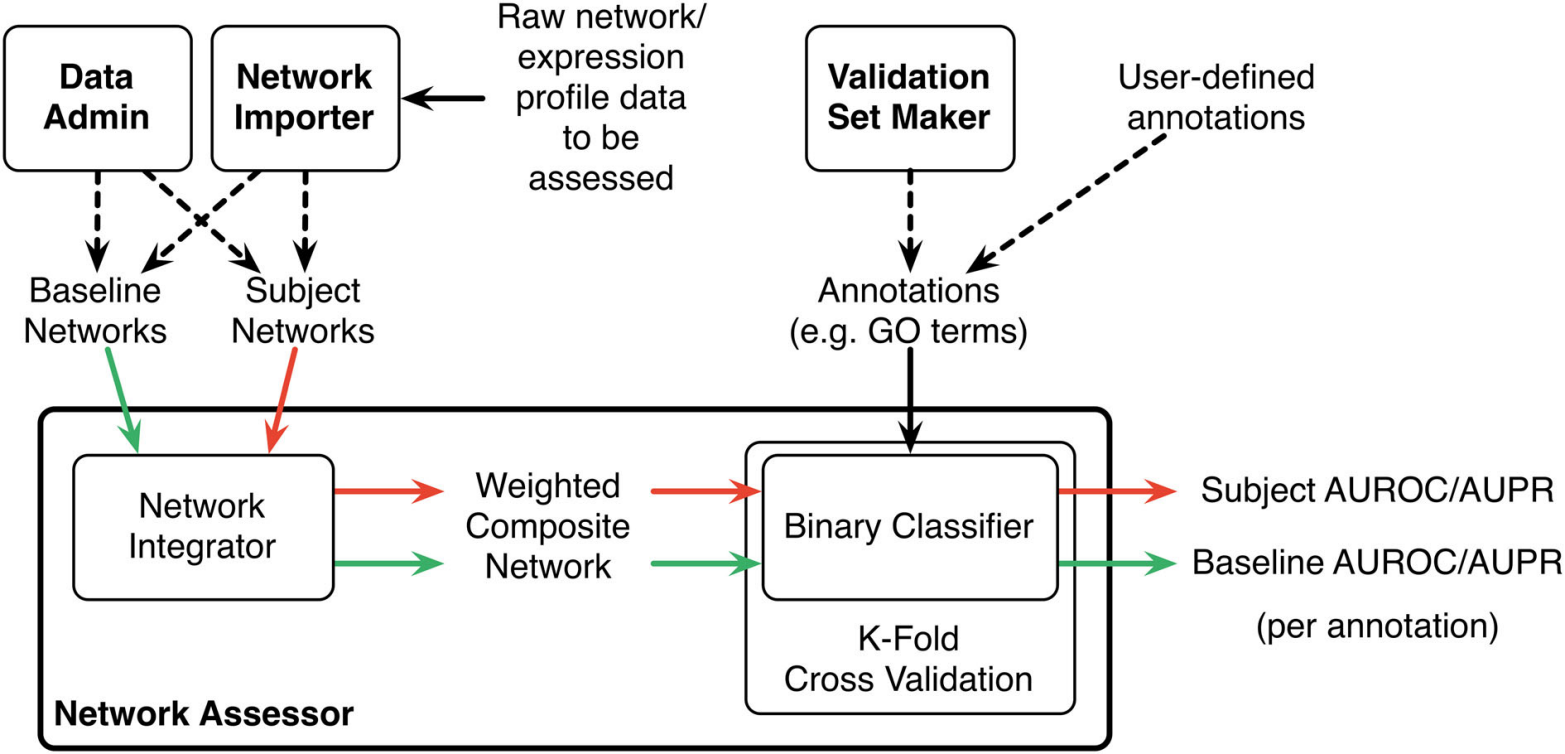
**Schematic diagram of the Network Assessor workflow**. The green arrows indicate the first round of cross validation. Red arrows are second round. AUROC/AUPR statistics are computed for each node label (e.g., GO term) for each round of cross validation. Dashed lines indicate alternative options.

Network Assessor measures the predictive potential of an association network by following a five step process (Figure 1). First, the set of baseline networks are combined into a weighted, undirected graph using GM's “automatic” network integration algorithm (Zuberi et al., 2013). Specifically, we use the GO Biological Process (BP)-based Simultaneous Weights algorithm for queries with less than five genes, and the Unregularized algorithm for five or more genes (Mostafavi and Morris, 2010). This follows the default behavior of GM's network integrator.

Second, this composite network is used during K-fold cross validation to recover the annotations in the user-provided gold standard, such as a set of GO terms and the lists of genes they annotate. Annotated nodes are treated as positive examples and all others are treated as negative. The GM algorithm is used as the binary classifier during cross validation and an AUROC/AUPR statistic is computed for each annotation, for each fold. A perfect classifier produces AUROC and AUPR values equal to 1. A random classifier achieves AUROC equal to 0.5 and AUPR close to P/(P+N) where P and N are the number of actual positive and negative examples, respectively (Schrynemackers et al., 2013).

Next, Steps 1 and 2 are repeated using the subject (i.e., non-baseline) networks. Typically, this comprises the baseline networks with the association network(s) of interest added (or removed, if the association network is part of the baseline).

Finally, the percentage differences of the AUROC and AUPR values are computed for each annotation. The results are sorted by these differences to highlight which annotations perform better or worse when predictions are made with or without the association network of interest. This method is a generalization of the leave-one-out analysis that we described in (Costanzo et al., 2010) to measure the contribution of individual genetic interaction datasets to our understanding of functional relationships in yeast.

The results produced by Network Assessor are reported as a table in tab-delimited text format. Although we describe in this protocol how to use Network Assessor with gene-gene associations to validate against GO annotations, Network Assessor is generic enough to be used directly with any type of network data and gold standard.

Network Assessor is bundled with over 1800 GM networks for eight different model organisms. However, it is possible to use any organism and networks by using the Id Importer tool documented at http://pages.genemania.org/tools/.

### Materials

#### Prerequisites

- Familiarity with the Windows, Mac OS X, or Linux command line.

#### Reagents

- An association network in tab-delimited format. The first two columns are the identifiers of the interactors. These can be a mix of gene symbols, UniProt accessions/IDs, Ensembl Gene/Protein IDs, RefSeq mRNA/Protein IDs, TAIR IDs or Entrez Gene IDs. Optionally, a third column can be added to indicate the weight of the interaction. Here is an example weighted network with two interactions. “<TAB>” denotes a tab character, which must not be surrounded by spaces in the network file:

BRCA1<TAB>RAD50<TAB>0.25
BRCA1<TAB>MRE11A<TAB>0.34

#### Equipment

- A computer with at least 8 GB of RAM and an internet connection.

#### Equipment setup

The following software is required:

- Windows XP 64-bit, Mac OS 10.6 or Ubuntu Linux 8.04 64-bit (or equivalent) or later.
- Java 1.6 64-bit or later.
- GM command line tools version 3.3 or later (http://pages.genemania.org/tools/).

#### Procedure

Steps 1–3: Set up the command line environment.

1. Create a new directory for storing the results of your work.
2. Copy the GM command line tools JAR file into this directory. In later steps, we will assume this file is called genemania.jar. Also copy your association data into this directory.
3. In your shell, set the current directory to the working directory you just created.

Steps 4–10: Install baseline association data for your organism of choice.

4. Run the following command in your shell to list the available baseline data sets (The full documentation for this command and the ones below is available at http://pages.genemania.org/tools):

java -jar genemania.jar DataAdmin list
  The following shows the output of this command:

Data Set ID Total Size Database Version
2013-10-15 9351.08 MB 15 October 2013
2013-10-15-core 2059.38 MB 15 October 2013
2013-10-15-open_license 9324.49 MB 15
  October 2013
2012-08-02 5994.14 MB 19 July 2012
2012-08-02-core 1764.09 MB 19 July 2012
2012-08-02-open_license 5963.38 MB 19
  July 2012
…
5. Note the “Data Set ID” (first column) of the data you wish to install. The latest release as of this writing is “2013-10-15” which corresponds to database version 15-Oct-2013.
6. Run the following command to download the base data set. This should take no more than a few seconds using a 3 Mbit/s connection.

java -jar genemania.jar DataAdmin \\
install 2013-10-15
7. Run the following command to list the available organisms for the selected data set:

java -jar genemania.jar DataAdmin \\
list-data
  Here is an example of the output:

Data ID Description Status
1 A. thaliana Arabidopsis (2494 MB)
2 C. elegans Worm (282 MB)
3 D. melanogaster Fly (792 MB)
4 H. sapiens Human (2361 MB)
5 M. musculus Mouse (2137 MB)
6 S. cerevisiae Baker's yeast (458 MB)
7 R. norvegicus Rat (576 MB)
8 D. rerio Zebrafish (248 MB)
8. Note the “Data ID” (first column)” of the organism you wish to install. For example, human data is “4.”
9. Run the following command to install the organism-specific baseline data set. Human data takes approximately 60 min to download and install using a 3 Mbit/s connection. In general it takes 10–70 min depending on the organism selected.

java -jar genemania.jar DataAdmin \\
install-data gmdata-2013-10-15 4
  Note: \\ indicates a line continuation and should not be included in the command.

Step 10: Download a gold standard for cross validation.

10. Run the following command to download the latest GO annotations and save it to a file. You can choose a particular GO branch, such as “bp” for biological process, “cc” for cellular component, or “mf” for molecular function, or “all” for everything. In the following example, the GO terms for taxonomy ID 9606 from the “bp” branch will be saved in the file “go-terms.txt.” By default, this command will download the annotations from the European Bioinformatics Institute GO MySQL server. It takes about 8 min on a 3 Mbit/s connection.

java -jar genemania.jar \\
ValidationSetMaker \\
--organism 9606 --branch bp \\
--query go-terms.txt
  Here are the taxonomy IDs of the organisms currently available in GM:

**Table:** 

**Organism**	**Taxonomy ID**
*A. thaliana*	3702
*C. elegans*	6239
*D. melanogaster*	7227
*H. sapiens*	9606
*M. musculus*	10090
*S. cerevisiae*	4932
*R. norvegicus*	10116

Step 11: Import the association data you want to analyze into your data set.

11. Run the following command to install your association data. This assumes your association data is stored in a file called “network.txt,” is for the organism with taxonomy ID 9606 (human), and will be saved with the name “network1” and categorized into group “group1.”

java --Xmx6G --jar genemania.jar \\
NetworkImporter \\
--data gmdata-2013-10-15 \\
--organism 9606 \\
--name "network1" --group "group1" \\
--filename network.txt

Step 12: Use Network Assessor to analyze the association data you imported.

12. To specify all GM networks as a baseline, use “coexp, coloc,gi,path,pi,predict,spd.” To measure the impact of your network in isolation not including the baseline networks, use “network1” for the “–network” parameter. To measure the impact of your network added to the baseline, use “coexp,coloc,gi,path,pi,predict,spd,network1” instead. The following example will assess your network in isolation, using 5-fold cross validation on four simultaneous processing threads with GO terms containing between 3 and 10 annotations, inclusive, and store the results in “go-terms.result.txt”:

java --Xmx6G -jar genemania.jar \\
NetworkAssessor \\
--data gmdata-2013-10-15 \\
--auto-negatives \\
--baseline "coexp,coloc,gi,path, \\
pi, predict,spd" --seed 1 \\
--threads 4 --networks "network1" \\
--organism 9606 --folds 5 --min 3 \\
--max 10 --query go-terms.txt \\
--outfile go-terms. result.txt \\
  Cross validation is a highly-parallelizable process since each annotation in the validation set is assessed independently. Network Assessor can automatically distribute the work across all cores of a multi-core system by specifying the number of threads to use. You can also partition “go-terms.txt” into multiple files and process each file on a different cluster node. Since Network Assessor is a memory- and computation-intensive program, ensure that at least 6 GB of RAM are free prior to starting the assessment. It takes an eight core 2.53 GHz Intel Xeon E5540 system approximately 24 s per line in “go-terms.txt” on average for the 15-Oct-2013 full human data set using eight processing threads. Since human is our largest dataset, using another organism or a subset of the networks will allow faster cross validation times. On a 10-node cluster of similar nodes, assessing the network against 1000 GO terms would take around 40 min of real time (6.6 h of CPU time).
  The “–min 3” and “–max 10” parameters instruct Network Assessor to only consider GO terms with at least 3 and no more than 10 annotations. This is important because binary classification algorithms generally perform worse with small GO terms. Using ranges 3–10 and 11–300 will give similarly sized partitions when used with the current GO database (see below for further explanation).
  The “–threads” parameter should be set to the number of physical cores on your computer. For example, use “4” for a single quad core processor. For a dual-processor system with eight cores each, use “16.”
  Using the same non-zero “–seed” ensures the results of different runs of Network Assessor are reproducible, as long as all parameters and inputs are the same. Otherwise Network Assessor will have slightly different results due to how the folds are randomized. Specifying a seed will guarantee the K-folds of the baseline and subject data sets are partitioned the same way.
  Here is a sample of the first four columns of Network Assessor's output:

**Table:** 

**QUERY**	**BASELINE-AUC-ROC**	**SUBJECT-AUC-ROC**	**%ERR-AUC-ROC**
GO:0000046	0.498133458	0.548483434	0.101077
GO:0000117	0.471654812	0.516121807	0.094279
GO:0000114	0.461791463	0.503638908	0.09062

The first column indicates the GO term used in the assessment. The second column is the mean AUROC of the baseline networks across the K-folds. The third is the mean AUROC of the subject networks, which in this example is the new network in isolation. Finally, the fourth column shows the % improvement in subject AUROC compared to the baseline, computed as follows:
  $$\%ERR_{AUROC}=\frac{SUBJECT_{AUROC}}{BASELINE_{AUROC}}-1$$
  In addition to these, the actual output file has similar columns for the AUPR and precision-at-10% recall statistics. If you run into any issues or have any questions, you can get in touch with the GM team at http://pages.genemania.org/contact/.

### Network assessor and GeneMANIA quality control

The dataset used by the GM gene function prediction server is updated on a regular basis. It performs real-time predictions for eight model organisms using over 530 million gene-gene functional associations organized into over 1800 networks. These associations are the edges of networks, which are weighted, undirected graphs, and come from numerous independent third-party sources. For example, co-expression networks are derived from gene expression profiles from GEO (Barrett et al., 2013); protein and genetic interactions from BioGRID (Chatr-Aryamontri et al., 2013); protein interactions inferred through orthology from I2D (Brown and Jurisica, 2007); pathway interactions from Pathway Commons (Cerami et al., 2011); and protein interactions from iRefIndex (Razick et al., 2008). Shared protein domain associations are derived from InterPro (Hunter et al., 2012) and PFAM (Punta et al., 2012). Identifiers and their metadata are sourced from Ensembl (Flicek et al., 2013).

Data imported from third parties can change without notice so each GM release reflects the state of those sources at a fixed point in time. For example, in an older data update, R6 (19-July-2012), cross validation results indicated a general drop in performance relative to the previous release, R5 (21-Dec-2011). This prompted further investigation, through which we discovered GM no longer recognized 10% (2344) of the human gene symbols that R5 supported. This was due to changes within Ensembl between R5 and R6 beyond our control as well as the conservative nature of GM's identifier mapping process. For instance, if a gene symbol is found to map to multiple distinct genes, that symbol is dropped to avoid ambiguity. When considering all identifiers that GM recognizes (>273,000), including Uniprot IDs and synonyms, the net loss was 3% (8196).

The set of recognized identifiers determines which associations are imported from the third-party sources. If at least one interactor in an association is not recognized, that association is not imported, so the loss of gene symbols led to a loss in interactions, including those that might indirectly connect two genes with retained identifiers. These changes affected prediction performance. We corrected this issue in the latest release, R8, which addresses the identifier mapping issues introduced in R6 and now outperforms both that and R5.

#### Default networks

The GM dataset is represented as a collection of weighted, undirected graphs. The human dataset contains 164 million edges organized into 395 networks. Of these edges, 156 million are co-expression. To ensure responsiveness and high availability for the GM web server, it is not practical to always use all association networks for each prediction. Instead, GM uses a semi-manually curated subset of networks by default. This includes all the networks described above except predicted interactions that are not inferred through orthology, and select co-expression networks. To determine which co-expression networks to include, all the default networks and all co-expression networks are combined using the GO BP-based Simultaneous Weights algorithm, which assigns each network a weight. The top 20 co-expression networks with the highest weights are selected for membership in the default set. This results in only 6.8 million co-expression edges retained. The number of edges across all default networks is 13.7 million, which is about 8% of the total.

#### Assessment of default networks

Network Assessor was used to assess the predictive potential of default networks of R6 in isolation vs. all networks in R6 using human data. The same was done for R8. Five fold cross validation was used in each case against GO BP annotations that were downloaded on 18-Jul-2013 from the European Bioinformatics Institute GO MySQL database mirror. Following the work of Mostafavi and Morris (2010), GO terms were grouped based on the number of genes annotated by each term since GO terms with fewer annotations tend to exhibit worse prediction performance. This resulted in two partitions with similar sizes: 3–10 annotations (*n* = 3239) and 11–300 (*n* = 3271). These results are summarized in Table 1. In general, the full set of networks consistently performs better than the default set; except for AUPR on GO terms containing 3–10 annotations, where both the R6 and R8 defaults have higher AUPR than the full. This is likely due to overfitting since each network is assigned a weight by the integration algorithm, and the full set of networks contains more than twice as many networks as the default set. Performance also increased for all measures in R8 compared to R6.

**Table 1:** **Median AUROC and AUPR for all networks in R6 and R8, as well as the default networks of each, respectively (bold indicates higher number per comparison)**.

	**R6**	**R8**	**R6 (default)**	**R8 (default)**
**GO TERM SIZE = 3–10**				
Median AUROC	0.650	**0.694**	0.627	**0.684**
95% CI	±0.316	±0.311	±0.334	±0.329
versus R6 (*p*-value)		^*^4.74 × 10^−82^	^*^1.41 × 10^−27^	
versus R8 (*p*-value)				^*^8.11 × 10^−10^
**GO TERM SIZE = 11–300**				
Median AUROC	0.871	**0.890**	0.857	**0.882**
95% CI	±0.217	±0.195	±0.246	±0.212
versus R6 (*p*-value)		^*^9.96 × 10^−258^	^*^5.68 × 10^−129^	
versus R8 (*p*-value)				^*^4.53 × 10^−35^
**GO TERM SIZE = 3–10**				
Median AUPR	0.012	**0.019**	0.019	**0.026**
95% CI	±0.349	±0.409	±0.343	±0.408
versus R6 (*p*-value)		^*^1.35 × 10^−28^	^*^1.34 × 10^−18^	
versus R8 (*p*-value)				^*^5.19 × 10^−19^
**GO TERM SIZE = 11–300**				
Median AUPR	0.185	**0.220**	0.181	**0.215**
95% CI	±0.412	±0.528	±0.415	±0.529
versus R6 (*p*-value)		^*^8.45 × 10^−256^	4.62 × 10^−1^	
versus R8 (*p*-value)				3.56 × 10^−2^

The Wilcoxon rank sum test was performed on the following pairs conditions: R6 versus R8, R6 versus R6 (default), and R8 versus R8 (default). The p-values for these tests are listed with statistically significant values (p < 0.01) marked with an asterisk.

**Figure 2:**
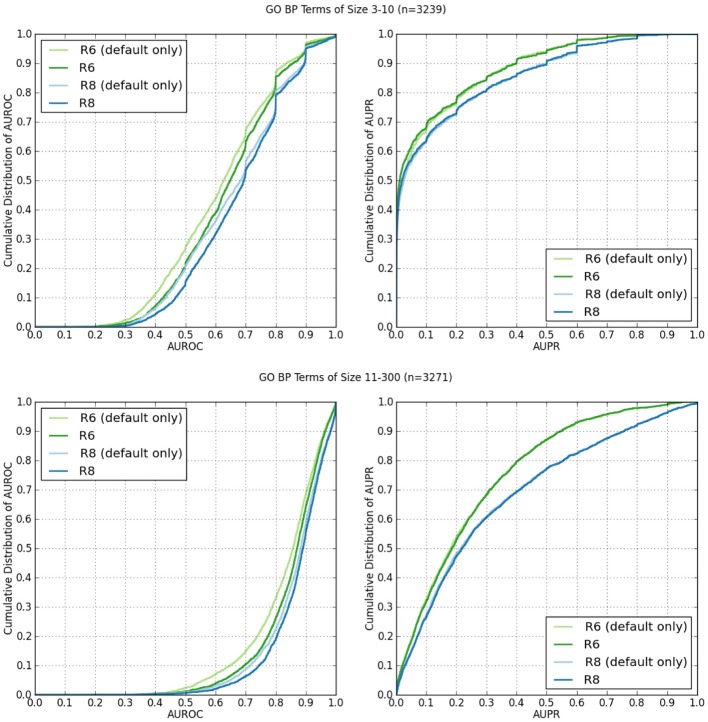
**Cumulative distributions of AUROC and AUPR of GO BP terms containing 3–10 annotations, and 11–300 from human network data from R6 and R8**. The “(default)” suffix indicates only the networks selected by default on the web server were used from the data set. The lack of the suffix indicates all available networks were used.

Figure 2 shows the cumulative distributions of the AUROC and AUPR of GO BP terms containing 3–10 annotations (*n* = 3239), and 11–300 (*n* = 3271).

Network Assessor was also used to analyze the relative predictive potential of default networks, as well as other key types of networks by measuring the degree to which prediction performance decreases when they are removed. Table 2 shows the AUROC and AUPR of GO BP terms containing 3–10 annotations, and 11–300 for R8 with default, co-expression, co-localization, genetic interaction, pathway, physical (protein) interaction, predicted, and shared protein domain networks removed, respectively. Median AUROC dropped by at least 4% when default or co-expression networks were removed. Median AUPR dropped by at least 14% when default, physical interaction, or shared protein domain networks were removed for terms containing 3–10 annotations. Median AUPR increased by almost 30% when co-expression networks were left out for the same terms. This is likely due to overfitting, which has been observed for smaller GO terms (Mostafavi et al., 2008). Median AUPR also increased by 3.4% when genetic interaction networks were left out for the same terms. In general, AUPR dropped by at least 15% when default networks were removed. AUPR also dropped substantially when predicted networks, most of which are derived through orthology from yeast, worm, fly, mouse, rat, were removed. This agrees with Klie et al. (2012) about the importance of intra-species transfer of annotations.

**Table 2:** **Median AUROC and AUPR for R8 when all networks are used (All) compared to when default (-default), co-expression (-coexp), co-localization (-coloc), genetic interaction (-gi), pathway (-path), physical (protein) interaction (-pi), predicted (-predict), and shared protein domain (-spd) networks are removed, respectively**.

	**All**	**-default**	**-coexp**	**-coloc**	**-gi**	**-path**	**-pi**	**-predict**	**-spd**
**R8**									
Total edges	1.64 × 10^8^	1.50 × 10^8^	6.94 × 10^6^	1.63 × 10^8^	1.59 × 10^8^	1.64 × 10^8^	1.63 × 10^8^	1.63 × 10^8^	1.63 × 10^8^
Edges removed from all	0	1.37 × 10^7^	1.57 × 10^8^	4.87 × 10^5^	4.85 × 10^6^	1.16 × 10^5^	2.75 × 10^5^	1.99 × 10^5^	1.02 × 10^6^
**R8: GO TERM SIZE = 3–10**									
Median AUROC	0.694	0.685	0.675	0.694	0.695	0.692	0.694	0.694	0.688
95% CI	±0.311	±0.309	±0.332	±0.311	±0.310	±0.311	±0.309	±0.310	±0.313
% difference from all		−1.3%	−2.8%	−0.1%	0.1%	−0.4%	−0.1%	−0.1%	−0.9%
versus all (*p*-value)		^*^2.17 × 10^−3^	^*^1.43 × 10^−23^	^*^5.04 × 10^−67^	^*^4.23 × 10^−109^	^*^1.46 × 10^−4^	^*^5.86 × 10^−13^	^*^4.05 × 10^−17^	7.05 × 10^−1^
**R8: GO TERM SIZE = 11–300**									
Median AUROC	0.890	0.866	0.864	0.889	0.890	0.887	0.887	0.890	0.880
95% CI	±0.195	±0.206	±0.224	±0.196	±0.195	±0.197	±0.199	±0.196	±0.199
% difference from all		−2.8%	−2.9%	−0.1%	0.0%	−0.3%	−0.4%	0.0%	−1.1%
versus all (*p*-value)		^*^0	^*^6.91 × 10^−183^	1.91 × 10^−1^	^*^8.24 × 10^−20^	^*^1.54 × 10^−22^	^*^1.01 × 10^−27^	8.83 × 10^−1^	^*^1.88 × 10^−219^
**R8: GO TERM SIZE = 3–10**									
Median AUPR	0.019	0.011	0.025	0.019	0.020	0.019	0.016	0.018	0.017
95% CI	±0.409	±0.377	±0.406	±0.409	±0.408	±0.407	±0.392	±0.411	±0.404
% difference from all		−44.8%	29.8%	0.0%	3.4%	−4.0%	−16.4%	−4.8%	−14.0%
versus all (*p*-value)		^*^2.43 × 10^−9^	^*^5.94 × 10^−15^	^*^6.98 × 10^−61^	^*^5.10 × 10^−83^	^*^8.71 × 10^−13^	^*^1.97 × 10^−11^	^*^3.40 × 10^−24^	^*^1.82 × 10^−4^
**R8: GO TERM SIZE = 11–300**									
Median AUPR	0.220	0.186	0.209	0.219	0.220	0.218	0.206	0.218	0.215
95% CI	±0.528	±0.527	±0.530	±0.528	±0.528	±0.525	±0.528	±0.528	±0.527
% difference from all		−15.6%	−5.0%	−0.4%	0.1%	−0.6%	−6.2%	−0.9%	−2.2%
versus all (*p*-value)		^*^1.14 × 10^−181^	^*^4.89 × 10^−9^	3.20 × 10^−1^	^*^3.63 × 10^−38^	^*^3.92 × 10^−16^	^*^1.20 × 10^−43^	^*^3.40 × 10^−24^	^*^1.82 × 10^−4^

The number of edges removed for each analysis is also listed, as well as total edges used during assessment. The Wilcoxon rank sum test was used to compute the listed p-values, where significant values (p < 0.01) are marked with an asterisk.

**Figure 3:**
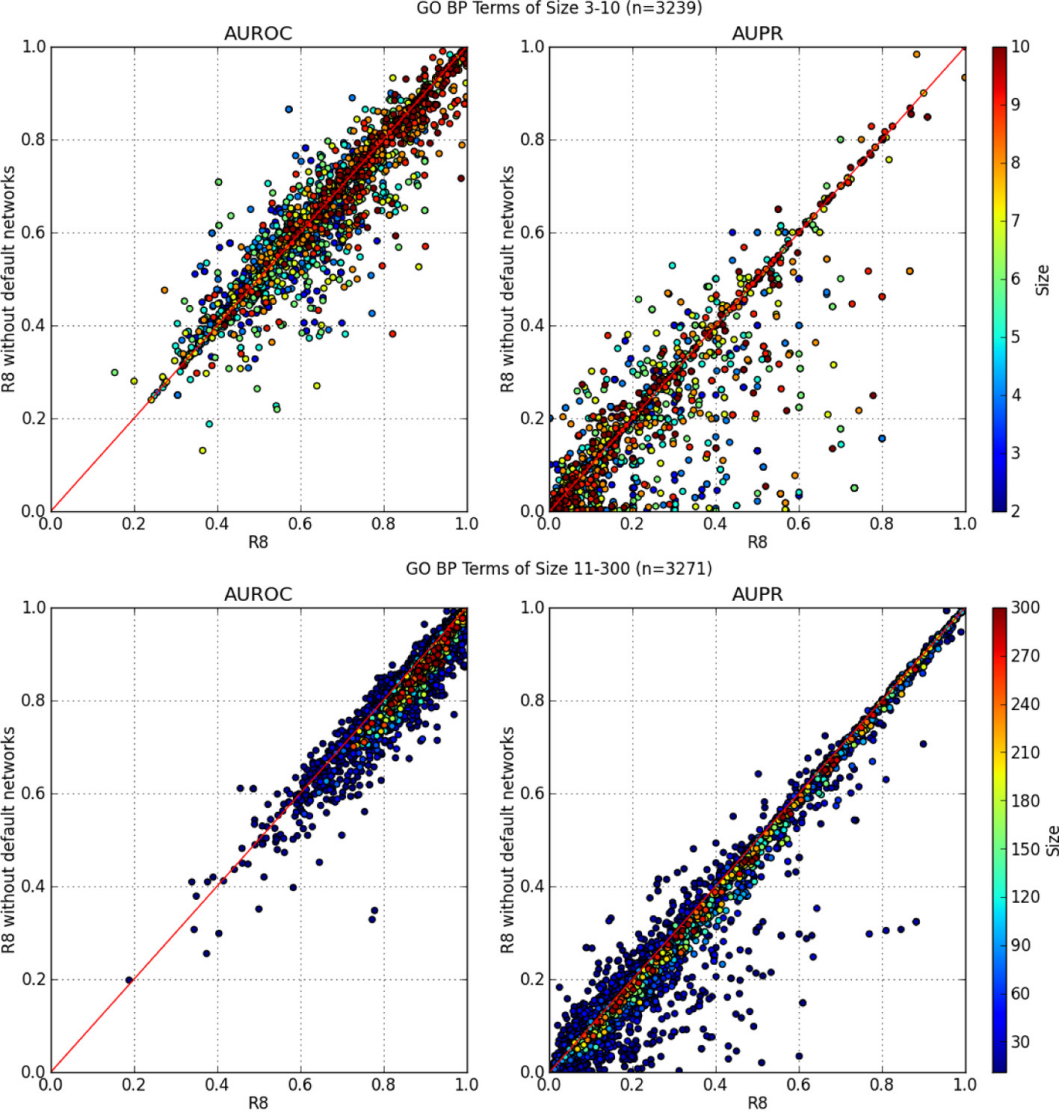
**AUROC and AUPR performance of each GO term**. X-axis denotes performance using all networks from R8 while the Y-axis is R8 without default networks. GO terms containing 90 genes or more consistently performed better using all networks from R8.

In Figure 3, the AUROC of most of the GO BP terms dropped when default networks were removed. The drop in AUPR was even more pronounced, regardless of the number of annotations in the GO term. This indicates an overall loss of precision and sensitivity when predictions were made without the default networks. The same analysis was performed for GO molecular function terms with similar results, which agrees with the findings of Mostafavi and Morris (2010).

## Conclusion

Network Assessor has proven useful for measuring the impact of the changes that occur in the third-party sources from which the GM prediction web server derives its training data and can be used by others for similar analysis with custom data. Network Assessor is open-source and is part of the GM project. Code is available on request, although migration to GitHub is planned.

### Conflict of interest statement

The authors declare that the research was conducted in the absence of any commercial or financial relationships that could be construed as a potential conflict of interest.
